# Invited Discussion on: A Meta-Analysis and Systematic Review of the Incidences of Complications Following Facial Threadlifting

**DOI:** 10.1007/s00266-021-02360-x

**Published:** 2021-06-01

**Authors:** Woffles T. L. Wu, Chin-Ho Wong, Bryan Mendelson

**Affiliations:** 1 Camden Medical Centre, Orchard Boulevard, Suite 09-02, Singapore, 248649 Singapore; 2 W Aesthetic Plastic Surgery. #06-28/29, Mount Elizabeth Novena Specialist Center, 38 Irrawaddy Road, Singapore, 329563 Singapore; 3 Centre for Facial Plastic Surgery, 109 Mathoura Rd Toorak, Melbourne, 3142 Australia

*Level of Evidence V* This journal requires that authors assign a level of evidence to each article. For a full description of these Evidence-Based Medicine Ratings, please refer to Table of Contents or online Instructions to Authors www.springer.com/00266.

The last two decades (1999-2021) can be regarded as “the era” for non-surgical facial rejuvenation by threadlifting. In that time we have seen the response to this innovative technique wax and wane with initial scepticism (1999), followed by guarded enthusiasm (2002), then an explosion of popularity (2003–2008), a period of disillusionment and decline in popularity as certain complications became apparent (2008–2011) and then a cautious re-acceptance of the technique again (2012–2017) and in recent years (2018–present), another explosion of enthusiasm as more and more products have become commercially available around the world and complications have diminished and results have improved.

During this time, good results, *cheek by jowl* with the suboptimal results, have shared the stage and this is understandable as threadlifting is not a single technique—it is many. There are different kinds of threads: some barbed, some with cones, some moulded, some short, some long, coiled, straight and a variety of other configurations, some monodirectional or bidirectional and some with attached needles and others that are inserted via a hollow needle introducer.

We have also witnessed an evolution and changing trend of the materials used for this assortment of threads, starting with non-dissolvable barbed monofilament polypropylene threads, followed by threads with a combination of polypropylene and resorbable cones, to barbed threads and cone threads made completely of dissolvable polydiaxonone (PDO) or poly L-lactic acid (PLLA). Wu and Mendelson have already commented on the logic behind the use of dissolvable threads [1].

This meta-analysis and systematic review of the complications of threadlifting is therefore timely [2]. However, we must be mindful that in any meta-analysis, culling from a multitude of papers that have already been written and may be very disparate or even conflicting in their reporting, the authors will be reliant only on what has been already published. This has inherent flaws as some studies list out complications that are peculiar to a particular type of thread or threadlift methodology being used and it may not be possible to extrapolate or generalise these findings. Nonetheless, we congratulate the authors of this current paper for a good quality, cohesive multi-study, multi-paper analysis of the most common threadlift complications capturing all the significant papers with the exception of one [3].

The authors of this paper state under Methods, their criteria for conducting the literature search, the keywords used, the inclusion criteria, evaluation of study quality, comparison of outcomes, data extraction and statistical analysis. Given the keywords searched included: rhytidoplasty, facial rejuvenation, face lift, barbed suture, threadlift, APTOS, suture suspension, silhouette suture, percutaneous and thread, we felt that the 2004 paper titled, Barbed Sutures in Facial Rejuvenation [4], written by the lead author of this commentary, Wu, may have proved useful to the meta-analysis had it been included.

This paper was one of the first papers published that addressed the role of barbed threads in facial rejuvenation and their complications. By way of history, Sulamanidze had authored the first and second papers on his invention, the APTOS barbed threads in 2000 [5] and 2001 [6], respectively, followed by a paper in mid-2004 on Lycka’s experience with 350 patients who underwent the APTOS procedure [7].

This was shortly followed by Wu’s 2004 paper, which described his experience with 102 patients who underwent the APTOS procedure and 112 patients who underwent the Woffles Threadlift. The Woffles Threadlift uses a patented bidirectional long barbed suture sling that spans the length of the jowls to the temple. In the Woffles Threadlift version 1.0 described in that paper, the bidirectional threads were inserted as a V-shaped sling where the apex of the folded thread is inferiorly in the cheeks and jowls whilst the free ends of the thread were tied superiorly in the temporal fascia of the scalp and buried subcutaneously. Both the APTOS and Woffles Lift threads are barbed and made of permanent monofilament polypropylene.

The complications of the Aptos treatments included: 11 palpable thread ends (10.8%), 8 migrations (8%), 5 infections or granulomas (5%) and 5 patients with dimpling and/or irregular waviness of the skin (5%).

The complications of the Woffles Threadlift version 1.0 were minor and included: 11 with knot palpability, knot exposure or granuloma in the scalp, requiring removal of the thread (9.8%) and 5 who had dimpling of the face that required release by subcision (4.5%). There were no infections that required removal of the threads.

With hindsight of these complications, Wu introduced the Woffles Threadlift version 2.0 [8–11] which inverted the suture sling such that the apex of the “V” was reversed and placed superiorly in the deep temporal fascia with the free ends of the folded thread located inferiorly in the cheek and jowl areas. No knots were tied in the scalp or face and this simple modification significantly decreased the complication rate to less than 2% (either extruded thread ends or protracted dimpling, both easily dealt with) with not a single thread infected nor removed.

In the current meta-analysis, the most frequent complications reported were: swelling (32.9%), dimpling (11.7%), paresthesia (6.8%), visible or palpable threads (1.8%), infection (3.1%), thread extrusion (3.9%) but the studies are not uniform nor were all the complications listed in all the papers.

We are of the opinion that it is not appropriate to lump together the complications of all the different types of threads used, to be interpreted as a whole, simply because of the diversity of thread types, design, materials and methodology. Certain complications may be peculiar to a particular thread. It is not possible to equate complications of permanent threads to the complications of composite or totally dissolvable threads even though on the face of it, they may appear similar. As such although the findings of this 26 paper meta-analysis are useful, caution is required in its final analysis and interpretation.

Furthermore, we take issue with what actually constitutes a complication and this has been commented on previously [12]. Like Sulamanidze has stated, we do not feel that swelling is a complication, as it spontaneously resolves, usually within two weeks. It should therefore be considered part and parcel of the procedure and not a sequelae. Swelling is only a complication when it becomes prolonged beyond a reasonable time for spontaneous resolution.

Similarly for dimpling, is this really a complication or is it again just intrinsic to the procedure? After all, we are essentially using specially designed internal threads to pull on soft tissue with no skin resection. It is inevitable that there must be some bunching or cinching of the overlying skin envelope or even visible traction lines. We note in this meta-analysis that the duration of dimpling was not analysed.

**Fig. 1:**
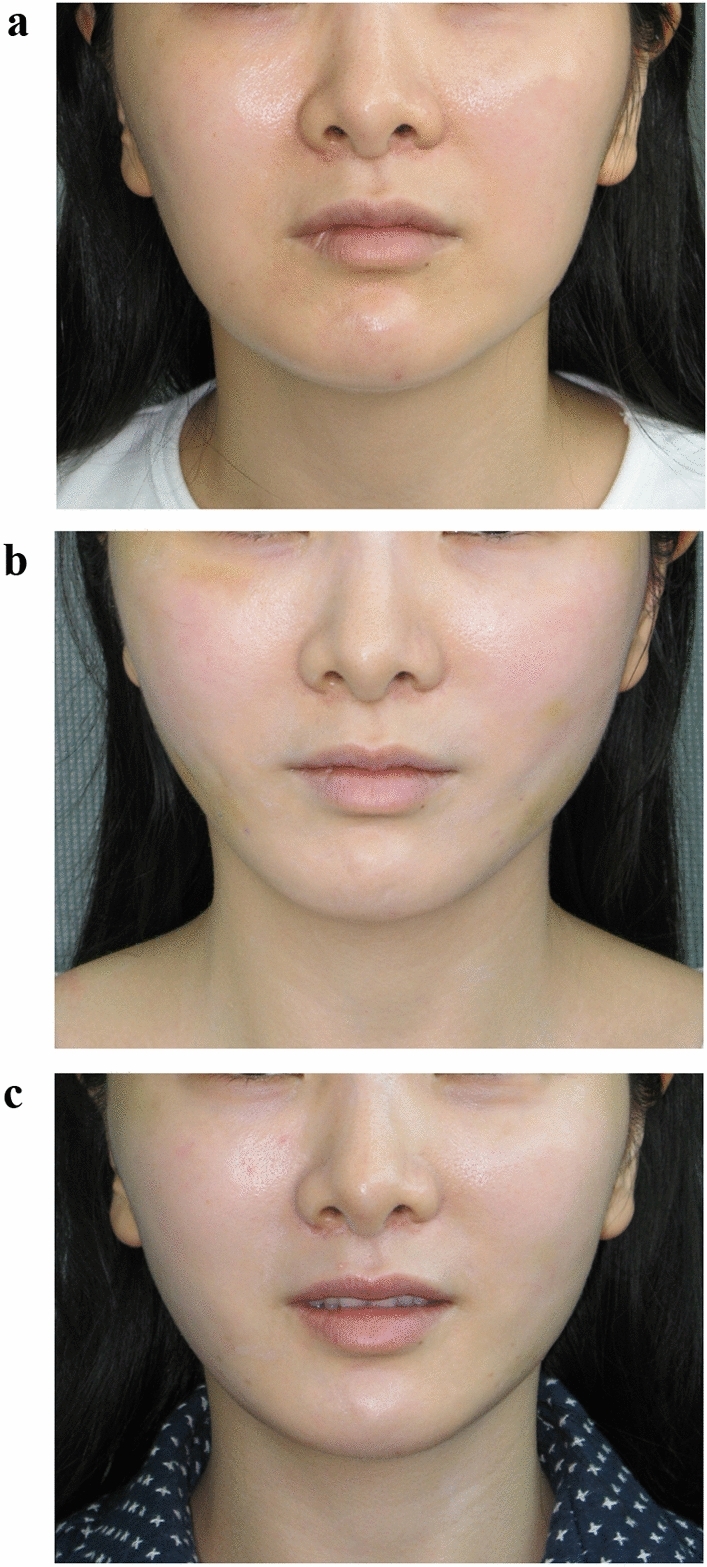
31-year-old East Asian female complaining of early jowl formation and laxity of the cheeks leading to an unpleasant, sulky appearance. She wishes to have a more youthful, pleasant countenance but rejects any open surgical procedures. **a** Preoperative (front view)—shows squaring of the lower face with cheek and jowl descent. **b** Postoperative 6 days—8 Woffles Threads were used on either side of the face, spanning the distance from the jowls to the temples. The procedure was performed only under local anaesthesia (6cc of lidocaine 2%/ 1:200,000 Adrenaline administered to each side of the face). Some bruising and swelling of the lateral face and cheeks with dents and undulations of both cheeks and mild traction lines can be seen. The jowls have been superiorly suspended and are no longer visible. **c** Postoperative 21 days—complete resolution of bruising, swelling and any dents or traction lines. The facial shape is sharper and appears lifted with eradication of visible jowls. The surface of the cheeks is smooth.

**Fig. 2:**
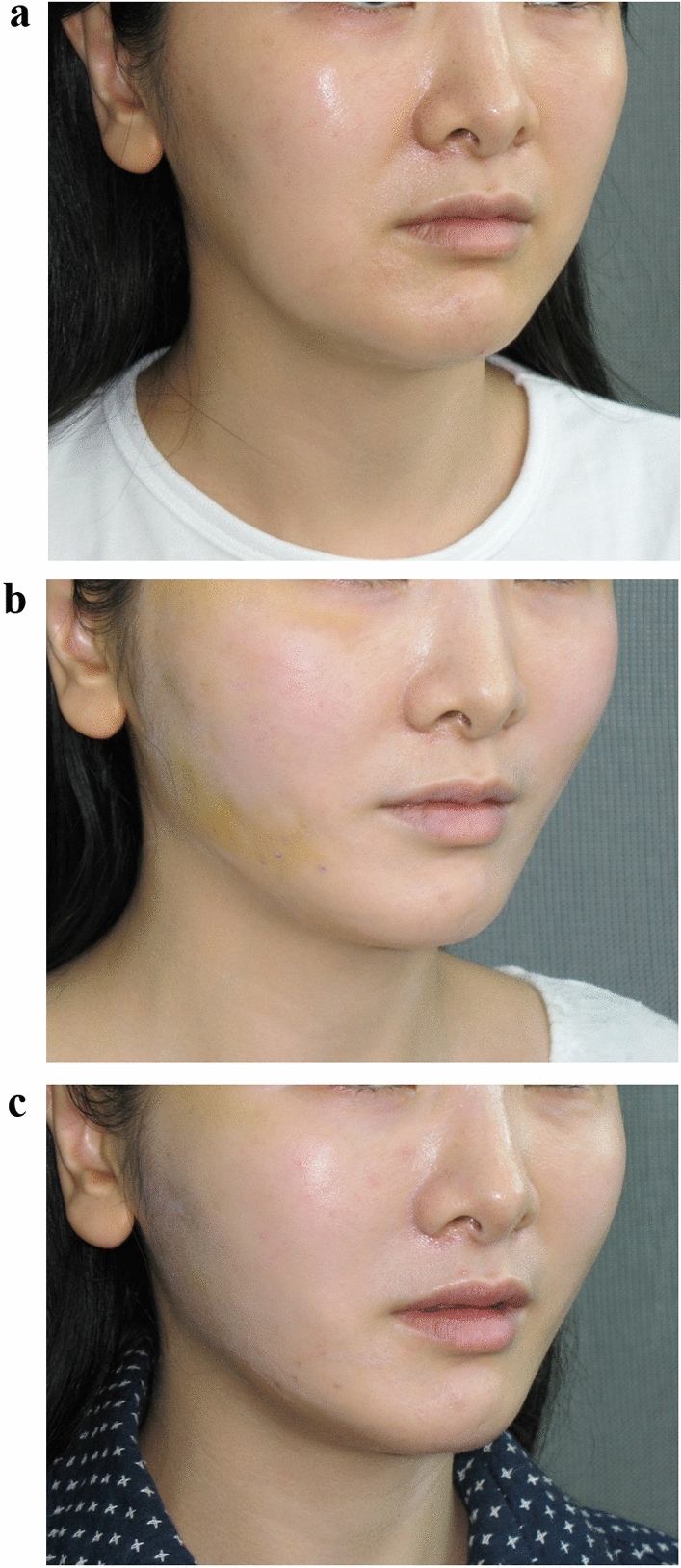
**a** Preoperative (front view)—the sagging jowls and convex contour of the face with an absence of a distinct Ogee line are apparent. **b** Postoperative 6 days—bruising, swelling, surface irregularities and denting with traction lines are seen. **c** Postoperative 21 days—a more pronounced and attractive Ogee curve of the face is apparent with elevation of the malar mounds and cheeks, eradication of the visible jowls, a narrower lower face and improved jawline. The facial contour is now more consistent with that of a younger face.

In the APTOS procedure or the Woffles Threadlift, every patient has some dimpling or surface undulations at the conclusion of the operation. If there is no dimpling at all, the result will neither be significant nor long lasting. Most of these dents will have ironed themselves out within a week or two (due to facial movements and mastication), failing which they may need gentle finger manipulation to massage free, the points of puckering. Rarely have we encountered dimpling beyond three weeks (See Figs. 1 a-c, 2 a-c).

If a dissolvable suture has been used, the dimpling will invariably disappear as the barbs or cones dissolve and the holding power of the thread diminishes. But if a permanent thread has been used and there is postoperative dimpling, then the time frame must be examined to see whether it constitutes a complication or not. If the dimpling spontaneously resolves within 4 weeks, it should not be considered a complication, but if the dimpling persists beyond that time and becomes visually unacceptable, requiring manual massage, manipulation or even a subcision, then this should be considered as a complication and duly recorded.
